# Effect of low-dose steroid on NF-κB and caspase-3 intestinal expression in a sepsis mouse model

**DOI:** 10.1186/cc10413

**Published:** 2011-10-27

**Authors:** HA Guntur, HP Diding, HT Pohan, D Widodo

**Affiliations:** 1 Indonesian Society for the Study of Tropical Medicine and Infectious Diseases, Surakarta, Indonesia; 2 Faculty of Medicine, Sebelas Maret University, Surakarta, Indonesia; 3 Indonesian Society for the Study of Tropical Medicine and Infectious Diseases, Jakarta, Indonesia

## Introduction

The use of low-dose corticosteroids in sepsis early stages is still debated. The association of LPS-LBP complexes to CD14 receptors and will interact with TLR4 to induce NF-κB as a signal and transcription of proinflammatory cytokines [1,2]. Excessive production of inflammatory cytokines will cause activation of SIRS, especially in gut-associated lymphoid tissues [3], which induces metabolic changes leading to apoptosis network, MOF, septic shock and death [3-5]. Changes in apoptosis are mediated by caspases, including caspase-3 that acts as an effector caspase [6,7]. Low-dose corticosteroids can inhibit the production of proinflammatory cytokines, production of inflammatory mediators, and lower adhesion of leukocytes to the endothelium [8].

## Objective

The aim of this study was to analyse NF-κB and caspase-3 intestinal expression, and also survival from use of low-dose steroid in the early stages of sepsis in the Balb/C mouse model of sepsis.

## Methods

Male Balb/C mice were inoculated with lipopolysaccharide for the sepsis mouse model. Sepsis mouse model grouping was to a sepsis group (Group I) and to sepsis with steroid (methylprednisolone 1 to 1.5 mg/kg BW/day) (Group II). Detection of intestinal NF-κB and caspase-3 expression used the immunohistochemistry technique on days 1, 3, 5 and 7. Survival was seen until the 7th day. The two-tailed Fisher exact test for the analysis of mortality, independent-sample *t *test for intestinal NF-κB and caspase-3 expression, and *P *< 0.05 were used to determine significant differences.

## Results

Acute inflammatory response occurs in the early stages of sepsis (the first 5 days of exposure) and the process of death occurs in advanced stages of sepsis (after the first 5 days of exposure) [9]. This study shows that the use of low-dose corticosteroids in sepsis early stages (first 5 days) significantly inhibited the expression of NF-κB (see Table 1), so cytokine production of proinflammatory cytokines was not excessive. Reduced product proinflammatory cytokines would reduce the expression of intestinal caspase-3 (see Table 2), which will reduce the excessive apoptosis in the intestinal tract. Decreased expression of NF-κB and caspase-3 in the intestinal tract would further reduce the excessive mucosal cell death. This situation will block the destruction and disruption of mucosal defense function of the digestive tract, thereby increasing immune response. The end result will be seen that low-dose corticosteroids can reduce mortality. This study found dead animals for Group I were 70%, while Group II were 10% (*P *= 0.020).

**Table 1:** NF-κB intestinal expression

	Amount of cell expression		
		
Day	Group I	Group II	*P *value
1	17.5 ± 4.7	10.8 ± 3.6	0.019
3	26.5 ± 4.4	15.8 ± 3.6	0.001
5	39.3 ± 4.1	31.2 ± 7.0	0.033
7	54.8 ± 9.6	50.5 ± 10.7	0.476

Data presented as mean ± standard deviation.

**Table 2:** Caspase-3 intestinal expression

	Amount of cell expression		
		
Day	Group I	Group II	*P *value
1	8.5 ± 2.9	4.3 ± 1.9	0.014
3	14.7 ± 3.1	9.8 ± 2.2	0.012
5	33.2 ± 8.3	12.8 ± 4.5	0.000
7	42.3 ± 3.2	37.7 ± 8.2	0.336

Data presented as mean ± standard deviation.

## Conclusion

Low-dose steroids can reduce NF-κB and caspase-3 intestinal expression and also mortality in early sepsis.
